# Phase transitions of the typical algorithmic complexity of the random satisfiability problem studied with linear programming

**DOI:** 10.1371/journal.pone.0215309

**Published:** 2019-04-19

**Authors:** Hendrik Schawe, Roman Bleim, Alexander K. Hartmann

**Affiliations:** Institut für Physik, Universität Oldenburg, Oldenburg, Germany; Scuola Internazionale Superiore di Studi Avanzati, ITALY

## Abstract

Here we study linear programming applied to the random *K*-SAT problem, a fundamental problem in computational complexity. The *K*-SAT problem is to decide whether a Boolean formula with *N* variables and structured as a conjunction of *M* clauses, each being a disjunction of *K* variables or their negations is satisfiable or not. The ensemble of random *K*-SAT attracted considerable interest from physicists because for a specific ratio *α*_*s*_ = *M*/*N* it undergoes in the limit of large *N* a sharp phase transition from a satisfiable to an unsatisfiable phase. In this study we will concentrate on finding for linear programming algorithms “easy-hard” transitions between phases of different typical hardness of the problems on either side. Linear programming is widely applied to solve practical optimization problems, but has been only rarely considered in the physics community. This is a deficit, because those typically studied types of algorithms work in the space of feasible {0, 1}^*N*^ configurations while linear programming operates outside the space of valid configurations hence gives a very different perspective on the typical-case hardness of a problem. Here, we demonstrate that the technique leads to one simple-to-understand transition for the well known 2-SAT problem. On the other hand we detect multiple transitions in 3-SAT and 4-SAT. We demonstrate that, in contrast to the previous work on vertex cover and therefore somewhat surprisingly, the hardness transitions are not driven by changes of any of various standard percolation or solution space properties of the problem instances. Thus, here a more complex yet undetected property must be related to the easy-hard transition.

## Introduction

The *Satisfiability problem* (SAT) [1] is to decide whether some Boolean formula is satisfiable or not, i.e., whether for a given Boolean formula, there is an *assignment* of the variables such that the formula evaluates to “true”. All Boolean formulas can be expressed in *conjunctive normal form* (CNF) which is a disjunction of *clauses*, each being a conjunction of variables or negated variables. Therefore *K*-SAT, which is a Boolean formula in CNF with *K* distinct variables per clause, is a commonly scrutinized version of the satisfiability problem.

NP-complete (nondeterministic-polynomial) problems [1, 2] are fundamental to computational complexity, since all problems in NP can be mapped in polynomial time to any NP-complete problem. Despite much effort, no algorithm has been found so far which is able to solve any NP-complete problem in the *worst case* in polynomial time, leading to the famous *P-NP problem*. Thus, all NP-complete problems are considered being *hard*, so far. Since 3-SAT is the prime example [3, 4] for an NP-complete problem, if one day someone found a fast algorithm for 3-SAT, one could efficiently solve *all* problems in NP, especially also all NP-complete problems. To advance fundamental science and help the cause to either prove or disprove that actually a fast algorithm can exist, one wants to understand the reason for the apparent computational hardness. One somehow empirical approach is to analyze actual (relatively) hard instances of problems. This has attracted interest beyond computer science and complexity theory in statistical physics [5–7] and correspondingly *random ensembles* of suitably parametrized problem instances and their *typical hardness* have been investigated.

In the physics community, the most prominent random ensemble for *K*-SAT consists of a set of *N* variables and *M* clauses. Each clause contains *K* distinct variables which are chosen randomly, and each variable appears negated with probability 0.5. Especially interesting to statistical physicists is that this problem ensemble exhibits a *phase transition* at some critical value *α*_s_ of the density *α* = *M*/*N* [8]. For a large number of variables at *α* < *α*_s_ almost all problems are satisfiable (also denoted as SAT), above *α*_s_ almost all realizations are unsatisfiable (UNSAT). The occurrence of similar phase transitions has been observed frequently for other random ensembles of NP-complete problems [5–8]. This incited strong interest in the *K*-SAT problem [9–12] and many other NP-complete problems among physicists, resulting in a plethora of articles applying methods from statistical mechanics to study these phase transitions in more detail [13–18].

While this SAT-UNSAT transition is certainly the most scrutinized in the *K*-SAT problem, there exist more transitions. For example 3-SAT, where SAT-UNSAT occurs at *α*_s_ ≈ 4.26 [9], shows a transition to chaotic behavior at *α*_*χ*_ ≈ 3.28 [19], i.e., using a continuous time deterministic solver [20] the trajectory will find the solution if one exists, but it will show chaotic transient behavior above this threshold resulting in increasing escape rates from attractors. This leads to a higher computational cost and can therefore be used as a measure of hardness. Furthermore, there exists a clustering transition at *α*_c_ ≈ 3.86 [11, 21]. This means that here the organization of the space of the exponentially many degenerate solutions changes from one big cluster (*α* < *α*_c_) of solutions which are connected in assignment space to a solution space (*α* > *α*_c_) which is fragmented into many non-connected smaller clusters, one says *replica symmetry* is broken above this threshold.

To examine optimization problems, usually algorithms like the *branch and bound approach*, *stochastic search* or *message passing* are used in the statistical-mechanics literature. These algorithms operate in the space of feasible assignments and approach the optimum solution from above. Here “optimum” means that the number of unsatisfied clauses is minimized (in the sense of MAX-SAT), i.e., eventually becomes zero if a satisfying assignment is found. Thus for general minimization problems these algorithms yield upper bounds until the true minimum solution is found. As empirically studying the computational hardness always tells something about a problem in conjunction with a specific algorithm, it is desirable to investigate different algorithms, in particular approaches which differ fundamentally. The operations-research literature often uses *linear programming* (LP) [2, 22], which operates for combinatorial problems outside the space of feasible solutions, i.e., fundamentally different from the above mentioned algorithms. Suitably enhanced LP techniques are often used for real-world applications since they are versatile and efficient, which means they run typically in polynomial time. For combinatorial problems, e.g., NP-hard optimization problems, the application of pure LP yields solutions which are not necessarily feasible, which here means non-integer-valued assignments to the variables, but which establish a lower bound on the objective. While this technique is typically not used for SAT, for other NP-hard problems like the traveling salesperson problem the best current methods are based fundamentally on LP [23]. Thus LP somehow approaches for minimization problems the true feasible and optimum solution from below (In this sense the analog solver of Ref. [19, 20] also operates outside the feasible region). Nevertheless, a key observation is that whenever LP gives a feasible solution, it must be the true optimum solution of the combinatorial problem.

Because of the complementary nature of LP and the approaches usually studied in the statistical mechanics literature, it seems to be a worthwhile endeavor to study LP in the context of statistical mechanics questions. To our knowledge, such studies of the behavior of LP in regards to phase transitions have only been conducted for the vertex cover (VC) [24] and the traveling salesperson problem (TSP) [25]. For these problems there exist regions in parameter space of the random ensemble, where feasible and optimal solutions can be found in polynomial time. For VC on Erdős-Rényi random graphs, the LP approach yielded solutions up to the percolation transition of the graph ensemble. Therefore, the problem is *easy* with respect to LP up to the percolation transition, and *hard* beyond. For LP improved with cutting planes, another easy-hard transitions occurs at the point of the onset of replica symmetry breaking [16], which here corresponds to the clustering transition of SAT. Note that this coincides with the percolation threshold for the *leaf-removal core* [26]. This is the point where one would not reasonably expect easy instances anymore. For TSP the easy-hard transitions detected by the LP approach coincided with structural changes of the optimal tour which can be intuitively understood as increases in hardness. Since for TSP many more cutting planes exist, which are not yet tested, it is conceivable to use this technique to find more and more easy-hard transitions this way and understand them, leading to deeper insight into the problem.

Since *K*-SAT is the archetypal NP-complete problem, we wanted to extend those promising results of the mentioned previous studies. We are not aware of any application of LP approaches to random *K*-SAT so far. Although *K*-SAT is by definition a decision problem and not an optimization problem, as we will show below, we found also some “easy-hard” transitions. Nevertheless, these transitions occur at clearly lower values of the parameter *α* than the clustering transition, in contrast to VC on random graphs. This result could be related to the fact that *K*-SAT shows a richer behavior [11], i.e., several different types of transition, in contrast to VC on random graphs. With the present study, we can exclude at least that these transitions are driven by a standard percolation (connectivity, pure-literal core, *q*-core, leaf-removal, and biconnected component) transitions on the underlying graph representation. However, since the graph representation should include all information, a property of the graph should change at this point, thus we think this study will motivate further studies which aim at the origin of the different behavior. On the other hand, this is a hint that the hardness of VC on random graphs is much clearer cut than for SAT. The difference could be due to the fact that the ensembles of random graphs exhibit different properties than the (bipartite) factor graph representation of random K-SAT.

Note that this study does not aim to present faster methods to solve the SAT problem, but rather tries to study it in a fundamental sense, aiming at the question “What makes a problem hard?” We pursue this by applying an approach which operates outside the space of feasible solutions and is widely-used for many practical combinatorial optimization problems, but less-often studied when considering *K*-SAT. In fact, we will show that the easy-hard transitions with respect to the used LP algorithms happen at rather low values of *α*. Thus, other more specialized algorithms are preferable for practical *K*-SAT solving.

## Models and methods

### *K*-SAT

A realization of *K*-SAT consists of a Boolean formula over *N* variables *x*_*i*_ (*i* = 1, …, *N*). The formula is in conjunctive normal form, i.e., it is a conjunction of *M* clauses *c*_*j*_ (*j* = 1, …, *M*), where every clause is a disjunction of *K* literals *l*_*kj*_ (*k* = 1, …, *K*). A literal is a variable *x*_*i*_ or its negation ${\bar{x}}_{i}$. In each clause, each variable may appear only once. As an example for *N* = 4, *M* = 2 and *K* = 3 take

$$\begin{array}{c} \left({\bar{x}}_{1}\vee x_{2}\vee {\bar{x}}_{3}\right)\wedge \left(x_{1}\vee x_{3}\vee {\bar{x}}_{4}\right). \end{array}$$ (1)

This example is solvable with, e.g., *x*_1_ = “true” = 1 and *x*_3_ = “false” = 0, and arbitrary assignments for the other variables. Note that each clause is satisfiable by 2^*K*^ − 1 out of 2^*K*^ possible assignments to the variables. Thus, each clause restricts the space of satisfiable assignments a bit. Clearly, with more clauses per variable, i.e., a higher amount of constraints, it is more probable that a random formula is unsatisfiable. As mentioned before, for random 3-SAT with *N* → ∞ there is a critical density *α*_s_ = *M*/*N* ≈ 4.26 [9] at which a phase transition from satisfiable to unsatisfiable (SAT-UNSAT) happens.

### Linear programming

A linear program (LP) is an optimization problem, which can be expressed by a set of linear constraints and a linear objective function, which should be optimized. There are fast (polynomial-time) algorithms to solve a linear program, e.g., the ellipsoid method [27] or interior point methods [28, 29]. However, in many sophisticated solvers the simplex algorithm is used, which typically terminates quickly for real-world problems, despite its exponential worst-case time complexity [2, 22]. Though, as soon as some variables need to be integer valued, this problem gets computationally hard. In fact, *integer linear programming* is an NP-hard problem [4].

A *K*-SAT realization can be expressed as an integer linear program. Therefore every positive literal *x*_*i*_ is expressed as an integer variable *x*_*i*_ and every negative literal ${\bar{x}}_{i}$ as (1 − *x*_*i*_). Since one or more literals of every clause *c* ∈ *C* need to be true for a satisfying assignment, the corresponding integer linear program contains for each clause the constraint that the sum of the expressions for the included literals must be greater or equal 1. The example from Eq (1) generates following linear inequalities.

$$\begin{array}{c} \left(1-x_{1}\right)+x_{2}+\left(1-x_{3}\right)\geq 1 \end{array}$$ (2)

$$\begin{array}{c} x_{1}+x_{3}+\left(1-x_{4}\right)\geq 1 \end{array}$$ (3)

Since an LP is an optimization problem but SAT is merely a decision problem, we can choose an arbitrary objective function for which to optimize. The simplest objective function is zero, i.e., no optimization.

$$\begin{array}{ccc} & \text{min}. & \;0 \end{array}$$ (4)

$$\begin{array}{ccccc} & \text{s}.\text{t}. & \sum_{x_{i}\in c_{j}}x_{i}+\sum_{{\bar{x}}_{i}\in c_{j}}\left(1-x_{i}\right)\geq 1, & & \forall 1\leq j\leq M \end{array}$$ (5)

$$\begin{array}{ccccc} & & x_{i}\in \{0,1\}, & & \forall 1\leq i\leq N \end{array}$$ (6)

The last constraint fixes the variables to integer values. We will relax this constraint to *x*_*i*_ ∈ [0, 1]. This allows us to apply a fast LP algorithm to solve the relaxed problem and introduce a measure of hardness for the problem realization. If the LP relaxation yields a solution consisting of only integer variables, the solution is obtained by a polynomial time method and the corresponding realization is obviously easy to solve.

A drawback is that additionally to the inherent degeneracy of the problem, i.e., there are possibly many assignments that satisfy the formula, the relaxation leads to a much higher degeneracy. For example, the assignment of all *x*_*i*_ = 0.5 is always a solution of the relaxation. After we performed some simulations for SAT in this way, it became evident that this degeneracy is a major problem for this decision problem, which is not present in the optimization problems studied with this method [24, 25]. This degeneracy leads to different behavior for slight changes in the algorithm. E.g., primal and dual simplex often lead to different behavior such that for many instances one version will result in an integer solution while the other does not. We observed a similar behavior when considering different pricing strategies or a presolve stage to tighten the LP. This analysis would therefore only yield information about these technical details and not about the fundamental problem of *K*-SAT. For example, the presolver of both Gurobi and CPLEX can solve easy instances up to an critical *α*_pre_ = 1.640(1), which is the same threshold up to which the *pure-literal* rule (also called *affirmative-negative rule*) which is an integral part of the DPLL [30, 31] search algorithm, can solve *K*-SAT realizations, while without presolve the easy-hard transition occurs at a lower value of *α*—dependent on technical details of the method. Therefore, we do not present results of LP with zero objective in is study.

Instead, we will introduce artificial objective functions to reduce the degeneracy drastically. Further, the choice of the objective function has an influence on the prevalence of integer solutions. Though note, only linear objective functions enable the efficient linear programming techniques. Therefore, non-linear objectives like ∑_*i*_ *x*_*i*_(1 − *x*_*i*_), which are minimal if all variables *x*_*i*_ are either 1 or 0, are not admissible and in fact generally NP-hard [32].

One simple way to replace the zero objective is maximizing the sum over all variables (MV)

$$\begin{array}{cc} & \text{max}.\;\sum_{i=1}^{N}x_{i}. \end{array}$$ (7)

While it might lift fractional variables to 1 and therefore proliferate integer solutions, it will also prefer variables to be fractional instead of 0 and thus suppress integer solutions. Since not a single fractional variable is allowed in an integer solution, one would expect this objective to only be able to solve some very simple instances and therefore not to be a good choice for an artificial objective, if one is interested in integer solutions.

Note that this and other additional objective functions have no influence on whether a formula is satisfiable or not, they are just meant as a tool to reduce the degeneracy of the problem to make it less dependent on details of the algorithm and to facilitate finding integer solutions. Both works out as we will see below. As a third objective we tried maximizing the number of fulfilled literals per clause, which we will call *Satisfaction Multiplicity Maximization* (SMM). This can be achieved with a slightly modified linear program by introducing one new variable *z*_*j*_ per clause counting the number of fulfilled literals of its clause and maximizing the sum over all *z*_*j*_.

$$\begin{array}{ccc} & \text{max}. & \;\sum_{j=1}^{M}z_{j} \end{array}$$ (8)

$$\begin{array}{ccccc} & \text{s}.\text{t}. & \sum_{x_{i}\in c_{j}}x_{i}+\sum_{{\bar{x}}_{i}\in c_{j}}\left(1-x_{i}\right)\geq z_{j}, & & \forall 1\leq j\leq M \end{array}$$ (9)

$$\begin{array}{ccccc} & & x_{i}\in \{0,1\}, & & \forall 1\leq i\leq N \end{array}$$ (10)

$$\begin{array}{ccccc} & & z_{j}\geq 1, & & \forall 1\leq j\leq M \end{array}$$ (11)

The new kind of constraint ensures that *z*_*i*_ ≥ 1, i.e., that every clause contains at least one fulfilled literal, such that the solution assignment satisfies the Boolean formula. This type of additional optimization is similar to MAX-SAT, where one tries to maximize the number of satisfied clauses. For MAX-SAT one would instead enforce 0 ≤ *z*_*j*_ ≤ 1 ∀*j*. We also tried this MAX-SAT approach to solve the *K*-SAT decision problem. Since this formulation does not mitigate the degeneracy problem, and because we did not observe any better performance than by using the other approaches, we do not show results for this approach here.

The SMM objective is an example for a linear objective with a slight preference for integer valued variables, since assignment of all variables of a clause to 1 or 0 according to their polarity contributes more to the objective function than assignments of non-integer values. For example a variable which appears more often unnegated will on average be assigned more often to value 1. This strongly reduces the degeneracy of the solution of the optimization problem, i.e., many of the solutions where the majority of variables are non-integer, are not optimal under this new objective function. Of course this may still yield non-integer values for some variables, which occur in conflicting clauses.

Note that when using LP, finding integer solutions may be facilitated in principle by adding cutting planes, which are further constraints which are added to the problem during run time dependent on the state of the solution process. This allows, e.g, to add a selected small number of constraints from a set of exponentially many ones, for which it would be infeasible to add them all. This was previously observed also for ensembles of random instances for the vertex cover [24] and the TSP [25]. Nevertheless, for the present study this yields no improvement, i.e., no additional phase transitions could be observed (see below). Thus, we do not describe the cutting-plane approach in this section in detail, but rather just list the the ones we tried and did not lead to an improvement.

### Mapping to vertex cover

**Fig 1:**
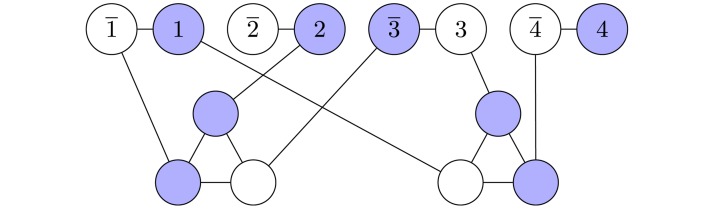
The graph for the vertex cover problem which is equivalent to the formula shown in Eq (1). Shown is a vertex cover of size *N* + (*K* − 1)*M* = 4 + 2 × 2 = 8, which corresponds to a satisfying assignment *x*_1_ = 1, *x*_2_ = 1, *x*_3_ = 0, *x*_4_ = 1.

All NP-complete problems, by definition, can be mapped onto each other in polynomial time. Thus, it is reasonable to ask, whether transforming SAT instances to instances of another problem and applying algorithms specifically suited for the other problem changes the performance, as measured by the location of the easy-hard transition. Here we used a classical mapping [1] of SAT to VC. For each *K*-SAT instance an equivalent graph *G* = (*V*, *E*) is constructed in the following way: The set *V* of nodes contains one pair of nodes $i,\bar{i}$ for each variable *x*_*i*_ (*i* = 1, …, *N*), which represents the variable and its negation. Furthermore, *V* contains one node (*kj*) for each literal *l*_*kj*_ (*k* = 1, ‥, *K*, *j* = 1, ‥, *M*) in each clause *c*_*j*_, respectively. Therefore *V* contains 2*N* + *KM* nodes. For the set *E* of edges, for each clause *c*_*j*_ a complete subgraph of size *K* is formed by connecting all pairs of “literal nodes” (*kj*) pairwise which correspond to this clause. Also, each “variable node” *i* is connected with its corresponding “negated variable node” $\bar{i}$. Finally, for each literal *l*_*kj*_, if the literal represents a non-negated variable *x*_*i*_, an edge connecting (*kj*) with *i* is included, while if the literal represents a negated variable ${\bar{x}}_{i}$ the corresponding literal node (*kj*) is connected with $\bar{i}$. Thus, *E* contains *MK*(*K* − 1)/2 + *N* + *MK* edges. Now a minimum vertex cover is obtained. This is a subset *V*′ ⊂ *V* of nodes such that for each edge of *E* at least one of the two endpoints is in *V*′. By construction [1], *G* contains a vertex cover of size *N* + (*K* − 1)*M* if and only if the corresponding formula is satisfiable. In Fig 1 the graph corresponding to the formula from Eq (1) is shown.

Thus, one approach to SAT is to transform each formula into the equivalent graph and use an existing algorithm for VC to solve it. We applied an LP formulation with cycle cutting planes, see Ref. [24] for details. For the previous work, this algorithm was able to solve VC instances in the parameter-space region, where the solutions were contained basically in one cluster, corresponding to the replica-symmetric region [16].

## Results

We sample random 3-SAT instances, where each clause may contain any variable at most once. For up to 12 system sizes *N* ∈ [64, 131072] we simulated *n* = 5000 realizations for 30 to 100 different values of the density *α*. For comparison, we also performed simulations for 2-SAT and 4-SAT, with a smaller range of sizes. All error estimates are obtained by bootstrap resampling [33–35], except for errors of fit parameters shown in the plots, which are *Gnuplot*’s asymptotic standard errors corrected according to Ref. [34]. To solve the LP realizations, the implementation of the dual-simplex algorithms of the commercial optimization library *CPLEX* [36] is used. During the research additionally the primal- and dual-simplex implementations of *Gurobi* [37] and *lp_solve* [38] with multiple pricing strategies were used to ensure that the results are independent from the algorithm and the details of the implementation.

### LP-Transitions with objective function

As mentioned before, we observed that non-trivial objective functions can be used to obtain a result independent from the details of the LP-solver implementation. Though the objective function itself will have an influence on the position of the transition point. An objective which prefers variables to be integer will result in more integer solutions at the same value of *α*, i.e., yield a transition at a larger value of *α*.

**Fig 2:**
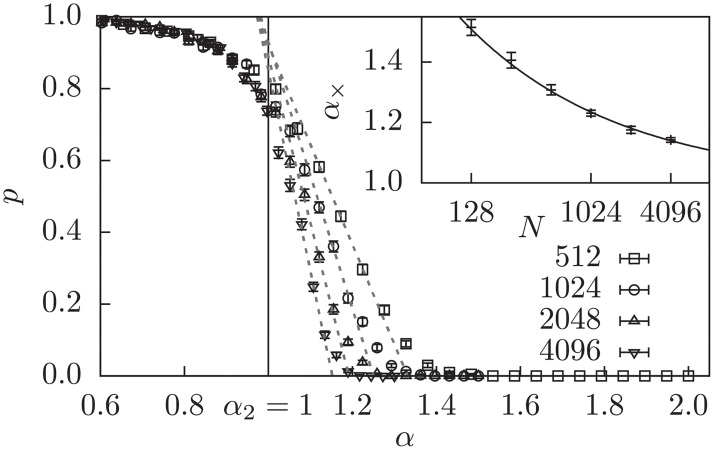
Solution probability *p* that SMM yields an integer solution for 2-SAT. The tangents at the inflection points are shown as dashed straight lines. Each tangent intersects the *α*-axis at some point *α*_×_ = *α*_×_(*N*) (see text). Inset: Extrapolation of the intersection points using the power law *α*_×_ = *aN*^−*b*^ + *α*_2_ This estimates *α*_2_ = 1.00(3) as the transition point, shown as vertical line in the main plot.

First, we will demonstrate that this method can be used to solve instances of *K*-SAT efficiently for a range of values of *α*, and that there exists a phase transition to a hard (or unsolvable) region. For the 2-SAT problem, which is not NP-complete, it is known that the SAT-UNSAT transition happens at *α*_2_ = 1 [39]. For 2-SAT there are also exact polynomial time algorithms. So when applying our linear programming approach, we would assume that in the limit of large *N* for *α* < 1, all instances are easily solvable and for *α* > 1 not. This test does indeed work out when using the SMM objective function. In Fig 2 the probability that a realization is solvable by the LP+SMM approach, i.e., the solution is integer, is shown as a function of the clause density *α*. Around *α* = 1 the behavior switches from solvable to not-solvable. The decrease in probability to solve is steeper for larger system sizes *N*, which is a behavior typical for phase transitions. To estimate the position of the phase transition in the thermodynamic limit of *N* → ∞, we extrapolate our results for finite system sizes to this limit. Often, the solution-probability curves as a function of the control parameter taken for different system sizes *N* intersect at some point, yielding an easy estimate of the transition point. This is not the case here. Instead, the measurements for small values of alpha seem to be independent of size *N* as visible in Fig 2. Thus, we assume that the asymptotic curve will coincide onto the system-size independent part and drop rapidly to zero at the transition point, similar as observed for the SAT-UNSAT transition in 2-XORSAT [40]. To estimate the transition point we take of each system size the inflection point, i.e., the point where the curve is steepest, and measure the intersection *α*_×_(*N*) of its tangent with the *α*-axis. In the asymptotic case, the inflection point should move downwards and the slope become steeper, such that extrapolating *α*_×_(*N*) for large *N* should give an estimate for the transition point *α*_2_. As a simple guess for the extrapolation function, we choose a typical finite-size scaling power-law ansatz *α*_max_ = *aN*^−*b*^ + *α*_2_, like in previous work [24, 25]. This ansatz does fit remarkably well as shown in the inset of Fig 2. This yields a critical point of *α*_2_ = 1.00(3), which is in very good agreement with the expectation, especially considering the small system sizes used for this extrapolation. Note that without the objective function 2-SAT is also susceptible to the greater degeneracy and the above mentioned problems become visible, leading to no clear result (not shown here). This result for 2-SAT shows that the method per se is a valid approach to our question.

**Fig 3:**
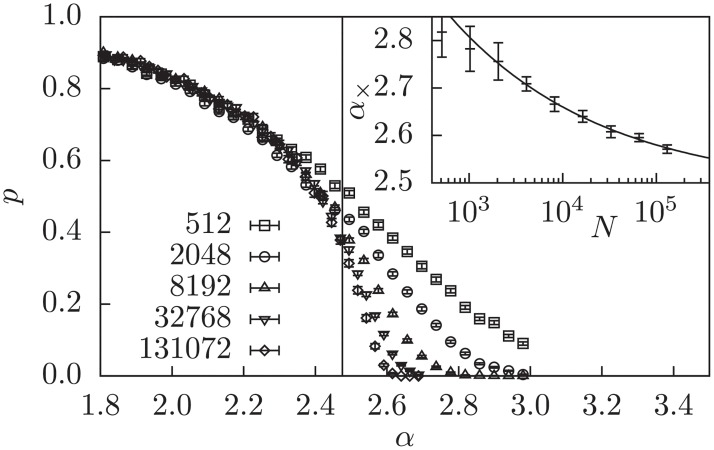
Probability *p* that SMM yields an integer solution. The smaller system sizes show visible deviations from the common curves, which is visible in the main plot, where *N* = 512 does not lie on the other curves at *α*_SMM_ marked by the vertical line. Inset: Extrapolation with the power law *α*_×_ = *aN*^−*b*^ + *α*_SMM_ of the intersection of the tangents at the inflection point with the *α*-axis (see text). This estimates *α*_SMM_ = 2.48(13) as the transition point.

The same procedure is performed for 3-SAT with the SMM optimization in Fig 3 yielding *α*_SMM_ = 2.48(13). The transition depicted here is an algorithmic transition from *easy*, since most realizations are solvable by LP techniques, i.e., in polynomial time, to some *harder* phase, where the LP does not yield solutions.

Our results for 4-SAT look similar (not shown) and exhibit an easy-hard transition as well. We performed a corresponding analysis. The resulting exponent *b* seems to be larger and a fit through the positions of the intersections of the inflection-point tangents yields *α*_SMM_ = 4.09(11).

The other optimization function of this study, MV, i.e., maximizing the sum of all variables, leads to a qualitatively similar behavior for 3-SAT as SMM but a transition at lower *α*_MV_ = 1.5(1) (not pictured due to qualitative similarity, simulations used smaller system sizes). The lower transition point is plausible, since this maximization prefers variables to be larger than zero instead of zero. For this reason, we have not analyzed this algorithm for 4-SAT. Our estimates for the transition points are collected in Table 1.

**Table 1:** Values of critical points. *α*_VC_ denotes the critical point when mapping SAT to VC and applying an LP + cutting plane solver used for VC. *α*_MV_ is the easy-hard transition for LP+MV. *α*_SMM_ is the easy-hard transition for LP+SMM. *α*_c_ denotes the clustering transition and *α*_s_ the SAT-UNSAT transition.

*K*	*α*_VC_	*α*_MV_	*α*_SMM_	*α*_IR_	*α*_c_	*α*_s_
2	–	–	1.00(3)	–	–	1
3	0.90(3)	1.5(1)	2.48(13)	2.98(3)	3.86	4.26
4	–	–	4.09(11)	–	9.547	9.93

Generally, one can even use a fractional LP relaxation to arrive at solutions. The most flexible and most important method for this are *cutting planes* (CP). These are further constraints which are added to the problem to cut off non-integer solutions from the polytope defined by the linear program. While there are exponentially many of these constraints, often only very few have to be enforced to arrive at an integer solution. In similar studies on VC [24] and the TSP [25], the introduction of cutting planes yielded substantially better results. For vertex cover the introduction of (potentially exponentially many but actually few) CPs even lead to an LP+CP transition at the point where in the analytic solution replica symmetry breaking, i.e., clustering of solutions appeared [16]. As shortly mentioned above, we also implemented cutting planes. Unfortunately, this efficiency of CP was not observable during our study for *K*-SAT. It seems that the cutting planes we tried, namely *resolution cuts* [41] and *clique cuts* [42], were too weak at the low values of *α* examined here. Another cutting plane for the SAT problem, the *odd cycle inequalities* [43] are not directly applicable for *K*-SAT with *K* ≥ 3, since they need clauses with 2 variables to be constructed. While they are useful as local cuts in a branch and cut procedure, they are never violated in the beginning for *K* ≥ 3 and thus not applicable for this study.

**Fig 4:**
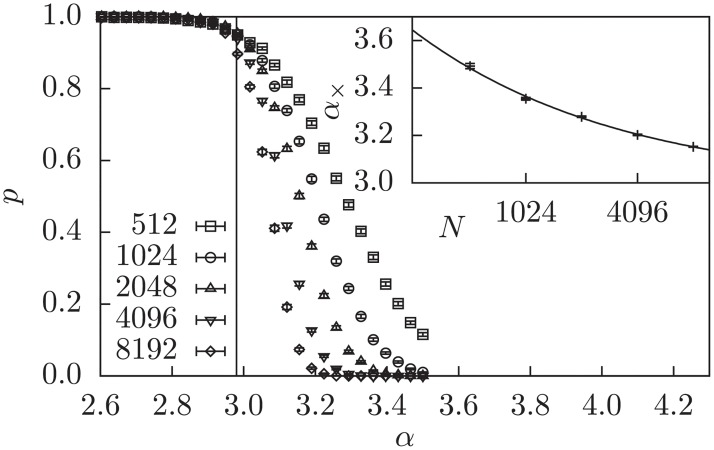
Probability *p* that SMM+IR yields an integer solution. Inset: Extrapolation with the power law *α*_×_ = *aN*^−*b*^ + *α*_SMM+IR_ of the intersection of the tangents at the inflection point with the *α*-axis (see text). This estimates *α*_SMM+IR_ = 2.98(3) as the transition point.

Another strategy to use information of the LP relaxation to arrive at an integer solution is the rounding of variables. Note that this strategy may lead to wrong assignments of variables, such that a realization which is satisfiable might become unsatisfiable after a variable is fixed to the wrong value. We found that the rounding of all fractional variables at once will not lead to significantly different measurements than without rounding (not shown). Instead we use a iterative rounding strategy (SMM+IR), which rounds in each iteration the variable closest to an integer and solves the LP, in which this variable is fixed. This is iterated until all variables are integer or the solution is infeasible, i.e., a contradiction is encountered. Fig 4 shows the solution probability of this approach, which leads to a transition at *α*_IR_ = 2.98(3). Interestingly, this yields far better results when optimizing for the SMM objective than with the pure LP, where we detected only very slight, and still on the solver dependent, improvement (not shown). Note that this protocol is still polynomial time since at most *N* iterations can take place, before all variables are fixed to integers.

Other general schemes to arrive from an LP relaxation at an integer solution rely on techniques with exponential worst-case time complexity, e.g., the backtracking-based branch-and-cut approach, where a search tree is build, where at each node a variable (in the case of SAT) is fixed to either 0 or 1 and new globally or locally valid cutting planes may be introduced at each node.

### Structural transitions

**Fig 5:**
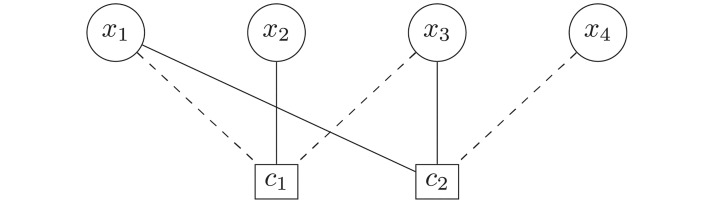
Factor graph representation of the example Eq (1). Dashed lines represent negative weights, corresponding to negated variables.

Next, we investigate whether the observed easy-hard transitions correspond to changes of the structure of the problem instances, as it was previously found for the vertex-cover problem [16–18, 24]. While VC is defined on graphs anyway, for *K*-SAT we study the related graph representation, the *factor graph* (FG) [6, 44]. This representation is especially useful for *belief* or *survey propagation* approaches [45, 46] but also useful to study structural properties. In the FG, which is a bipartite graph exhibiting a node for each variable and a node for each clause, i.e., containing *N* + *M* nodes, each variable is connected to the clauses it occurs in with weights representing whether they are negated or not. The corresponding graph of example Eq (1) is shown in Fig 5. Note that this representation, when disregarding the weights, is equivalent to a hypergraph representation, where every clause is represented by a hyperedge connecting *K* variables.

We looked at some known points of the factor graph, where its properties change, which could plausibly influence the hardness of the problem. Note that for the NP-complete VC, it was possible to relate the percolation threshold and the threshold, beyond which the leaf removal heuristic [26] does not yield solutions anymore (which coincides with the appearance of replica-symmetry breaking and solution-space clustering), to the points where the problems turns harder also for special formulations of the linear programming approach [24]. We will list the properties for 3-SAT we looked at. Most transitions are known from the literature, but few are not, to our knowledge. For those we numerically investigated the transitions during this study and present the result shortly here, which also contributes to the characterization of the 3-SAT ensemble.

- The *percolation threshold*, i.e., the point below which there will be no connected component of size O(N) is at *α* = 1/6 [6]. This means that below this threshold clauses do typically not share variables and are therefore largely independent, i.e., it should be rather easy to solve for almost any algorithm.
- The transition where the remaining *pure literal core* is of order O(N), i.e., the value of *α*, beyond which the pure literal rule, which is an integral ingredient for the classical DPLL search, does not lead to solutions anymore is at *α*_pl_ = 1.636‥ [47]. The pure literal rule is to remove pure variables and their clauses from the problem. Variables are *pure*, if they are appearing only in one polarity and can therefore be always set to fulfill all their clauses. Note that for 2-SAT the pure literal rule works up to the SAT-UNSAT threshold *α*_2_ = 1 [47], coinciding with the solvability transition of SMM.
- The *unit clause rule* shows its transition slightly above the SMM case but below the SMM+IR case at *α*_uc_ = 8/3 ≈ 2.66 [48].
- For a naive *q-core* analysis, where the core is the set of remaining nodes after all nodes with degree lower than *q* are iteratively removed, we treated the clause nodes the same as the variable nodes. It yielded a percolation transition, i.e., the existence of a *q*-core of order of graph size at *α*_2-core_ = 0.223(3) and *α*_3-core_ = 1.554(1) from our measurements.
- The *q-core* transitions for a hypergraph approximation of *K*-SAT, where *K* nodes are connected by single hyperedges, are known exactly. Note that the 2-core for this ensemble is equivalent to the pure literal rule according to Ref. [47]. The appearance of a 3-core occurs at *α*_3-CORE_ = 4.2847‥ [47].
- The *leaf removal rule*, which is a valid heuristic for the related XORSAT problem and the vertex cover problem, is known to have a transition at *α*_lr_ = 0.81847‥ [49]. Interestingly, although not obviously related, this is half of *α*_pl_ of the pure literal rule.
- The transition where a *biconnected component* [22, 50] appears, i.e., a connected component, in which every pair of nodes stays in the same connected component if any other node is removed, happens at *α*_bi_ = 0.190(20). Similarly, the *bi-edge-connected component*, from which an arbitrary edge can be removed while staying connected, shows the transition at *α*_bi-edge_ = 0.211(7). This value is close to the appearance of our naive 2-core, which is plausible since a 2-core consists of biconnected components possibly connected by single edges.

**Fig 6:**
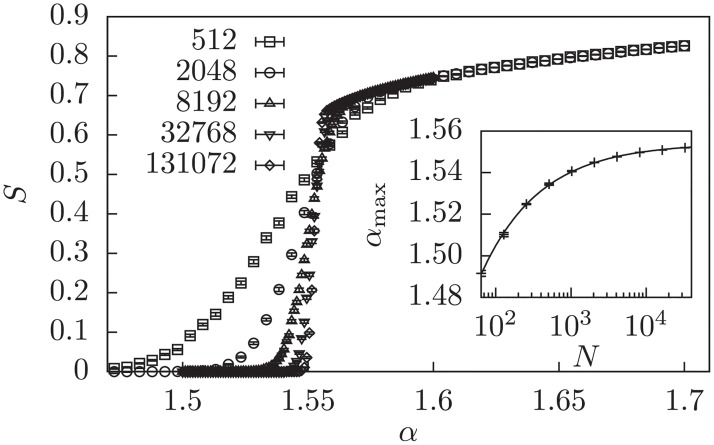
Relative size of the naive 3-core of the factor graph at different values of *α*. The inset shows the positions of the maxima of the peaks of the variance and an extrapolation according to a power law with offset, resulting in the above listed values.

The process to determine the transition points for the cases, where we did not find literature values, we use a similar process as for determining the transition for the LP variants before, but instead of extrapolating the intersections of the inflection-point tangents with the *α*-axis, we extrapolate the position of the maximum of the variance. This value can generally be determine more accurately and was already used in Ref. [24, 25], but seems underestimate the critical *α* for transitions of the form of Figs 2–4, where the left part is almost size-independent. An example for the naive 3-core on the factor graph is shown in Fig 6. Again we extrapolated the position of the peaks of the variance to large *N* using a power law with offset *α*_max_ = *aN*^−*b*^ + *α*_3-core_.

When comparing the values of these transitions to the easy-hard transitions points listed in Table 1, one observes no coincidence. Therefore, we can exclude that any of the observed transitions is driven by standard percolation transitions. Thus, in contrast to the previously studied VC, there exists no coincidence with a “simple” change of the problem structure, which might serve as an explanation of the observed easy-hard transitions. It appears that the easy-hard transitions are driven by a graph property, apparently by a non-standard one, which has yet to be identified.

Finally, the existence of correspondences between easy-hard transitions and structural percolation transitions for the vertex-cover problem motivated us to perform the following test: we also used for *K* = 3 the mapping of *K*-SAT to VC for formulas up to *N* = 10000. Using LP and cycle cutting planes [24] the corresponding equivalent VC instances were solved for various values of *α*. Again we measured the probability that an instance was solved by an integer solution as a function of *α*, for different system sizes. Using an analysis (not shown) as for the previous approaches, we were able to extrapolate an easy-hard transition for this point. We obtained a critical value of *α*_VC_ = 0.90(3), which is well below the easy-hard transitions obtained using the LP-based approaches presented above. Therefore, apparently it does not pay off using a mapping to another problem, at least for this pair of problems. A mapping to TSP will lead to TSP instances which are quite large for decent SAT realizations, therefore we did not perform simulations for this.

It is rather intriguing that SAT behaves so differently in comparison to vertex cover, despite their very close relation—both are NP-complete and can therefore be mapped onto each other. Nevertheless, the two random ensembles differ clearly from each other. While random graphs are locally tree like, i.e., exhibit only large loops, the representation of a *K*-SAT formula as a VC problem exhibits many short loops. Nevertheless, we not only have identified several easy-hard transitions in this study, but have also shed some light on this apparently fundamental difference of the two ensembles. In the future, maybe other approaches can yield a better understanding of this interesting fact.

## Conclusions

We study the solvability of random *K*-SAT realizations at different values of the clause-to-variable density *α* using linear programming. A realization is solved if the LP yields an integer solution. Since one can use LP algorithms that run in polynomial time even in the worst case, this means such a realization is “easy” in regard to this algorithm. Therefore it is sensible to assume that the structure of the typical instances changes at such a transition point, and this change is detected by the algorithm.

This study was mainly motivated by previous results for vertex cover [24], for which LP and LP combined with a simple class of cutting planes was able to detect increases in hardness, which coincided with easy to interpret transitions in the structure of the underlying graph. For vertex cover, this structural change could be tracked down and was found to be a percolation transition of the leaf-removal core. Furthermore, this transition coincides with the onset of replica-symmetry breaking, i.e., a complex organization of the solution space structure.

For random *K*-SAT, we were able to identify several such LP-based easy-hard transitions, to our knowledge for the first time. For *K* = 2 this transition is located right at the SAT-UNSAT transition, beyond which no algorithm can find a solution anyway. Interestingly, for *K* > 2 *K*-SAT does not behave as simple as VC, in that none of the transitions of different variations of the LP formulation coincides with an obvious percolation transition of the underlying graph structure. Also the transition to a clustered solution landscape, where replica symmetry is broken, can not be reached with any of the well known cutting plane classes for SAT. This complex behavior in regard to the LP hardness under scrutiny in this article, might be related to the more complex behavior of *K*-SAT anyway. This is true for example for the clustering behavior of *K*-SAT, where above the clustering threshold, other changes in the clustering behavior were detected, like the *freezing* transition at *α*_*f*_ = 4.254(9) [51].

A secondary result of technical nature we noticed, was the strong influence of a carefully crafted artificial objective function on the prevalence of integer solutions. We think it is worth to investigate this phenomenon further, as this might be useful when treating decision problems with an LP approach, like branch and cut. In our case we demonstrated that a crafted objective function can be more powerful than cutting planes, which are otherwise the main tool to reach integral solutions in an LP approach.

Notably, we detected for 3-SAT that an LP relaxation with the SMM objective can solve most realizations up to *α*_SMM_ = 2.48(13). With a straight forward deterministic iterative rounding scheme, it is possible to solve most realizations up to *α*_SMM_ = 2.98(3) in polynomial time.

We complement our data describing algorithmic easy-hard transitions by further results concerning the structure of graph representations of *K*-SAT. Some of these results cover different percolation transitions, which were not investigated beforehand. That none of the algorithmic transitions presented by us is coinciding with a simple structural property of the graph representation, despite our expectations set by results on the closely related VC and TSP problems, is in a way a negative result. However, since the hardness of a problem should be encoded in its structure, we think that a study examining more complex properties of the graph or any other representation, could yield insight into the reason for the failure of the LP approaches beyond certain values of *α*.

Inspired by our overall observation that the random *K*-SAT ensemble behaves differently than VC on random graphs with respect to the number of observed easy-hard transitions, future studies could examine whether the transitions which coincided for VC on Erdős-Rényi graphs will split into distinct transitions on graph ensembles more similar to the instances generated by the mapping from *K*-SAT, e.g., exhibiting many small loops. In general the influence of the ensemble on the type of observed phase-transition behavior is a wide-open problem, because for each of the so-far studied problems only one or very few “natural” ensembles have been investigated so far.

## Supporting information

S1 File Data. Data used to arrive all shown plots, fit values and qualitative statements.
(GZ) Click here for additional data file.
